# Rapid, sensitive, and specific, low-resource molecular detection of Hendra virus

**DOI:** 10.1016/j.onehlt.2023.100504

**Published:** 2023-02-10

**Authors:** N.M. Pollak, G.A. Marsh, M. Olsson, D. McMillan, J. Macdonald

**Affiliations:** a Centre for Bioinnovation, University of the Sunshine Coast, Sippy Downs, QLD, Australia; b DMTC Limited, Kew, VIC, Australia; c School of Science, Technology and Engineering, University of the Sunshine Coast, Sippy Downs, QLD, Australia; d CSIRO Health & Biosecurity, Australian Centre for Disease Preparedness, Geelong, VIC, Australia; e BioCifer Pty Ltd, Brisbane, QLD, Australia

**Keywords:** Hendra virus, N gene, Isothermal amplification, Recombinase polymerase amplification, Nucleic acid lateral flow, Nucleic acid extraction, Rapid test, Point-of-care, Nucleic acid amplification test

## Abstract

Efficient and accurate diagnosis of Hendra virus (HeV), a biosafety level 4 (BSL-4) pathogen and zoonotic disease, is of primary importance for surveillance and outbreak control in the Australian equine industry. Sporadic HeV spillover events pose a serious public health concern and are predicted to expand geographically, aligning with the moving distribution of the main reservoir hosts, the flying-foxes. Here we describe the development of a low-resource rapid Hendra test. The test used a fast and simple sample processing protocol followed by reverse transcription isothermal recombinase polymerase amplification (RT-RPA) combined with lateral flow detection (LFD) technology. Results were obtained in 30 min and required only a heating block, ice, and pipettes for liquid handling. The one-step sample processing protocol inactivated HeV in 2 min, providing a simple protocol that could enable safe testing outside of a laboratory. Analytical sensitivity testing demonstrated a detection limit of 1000 copies/μL of synthetic HeV RNA, and analytical specificity testing indicated assays did not detect other pathogens. Gamma-irradiated HeV-spiked in viral transport medium was detected with high sensitivity, down to 10,000 TCID_50_/mL, the equivalent of 18 RNA copies per reaction. Collectively, our data suggests that our rapid Hendra test offers a potential first-line screening on-site alternative to gold-standard RT-PCR detection, which requires samples to be shipped to central containment laboratories, thermocyclers and labour-intensive viral RNA purification, with testing time of approximately four hours. Our rapid Hendra test provided performance and speed without compromising sensitivity and specificity, and could become a promising more accessible tool for testing under resource-limited conditions for the veterinary community and thoroughbred industry.

## Highlights

• Development of a low-resource rapid molecular Hendra test.
• Rapid one-step sample processing protocol inactivating Hendra virus in 2 min.
• Test format using isothermal amplification lateral flow detection technology.
• Achieved detection of Hendra virus isolates in 30 min.
• Rapid Hendra test demonstrated 100% concordance with real-time PCR.

## Graphical abstract

**Figure:**
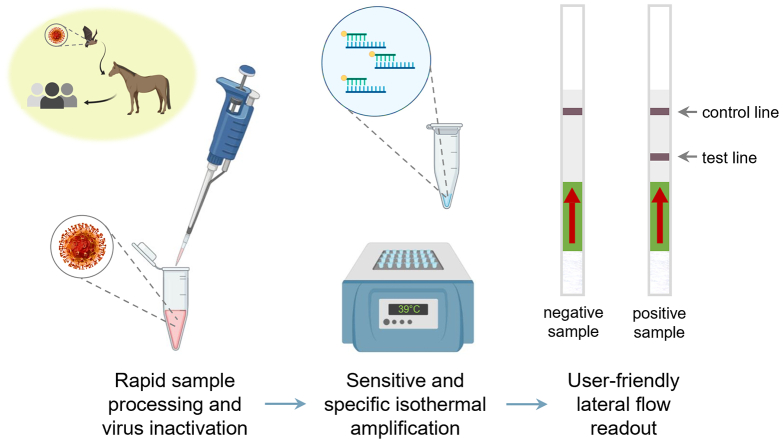

## 1 Introduction

Hendra virus (HeV) is a zoonotic pathogen of the *Henipavirus* genus within the *Paramyxoviridae* family. HeV has great epidemic potential because of its inherent characteristics it shares with other members of the *Paramyxoviridae* family (like measles virus), which are respiratory pathogens and transmitted through respiratory droplets. The virus is endemic in bats in Australia. Spillover events from this species are the primary source of equine infection where case fatality rates of 80% are reported [1,2]. To date there have been multiple spillover events in Australia, involving 107 horses, of which 87 cases have been confirmed [1]. These events mostly occur where urban and peri-urban areas are expanding and human population growth is high [3,4]. While HeV infections in humans remain rare, with only 7 reported cases [5], the mortality rate is 57% [6]. Transmissions to humans occurs directly from horses via contact with infected bodily fluids [7]. While there is a vaccine to prevent Hendra infection of horses, there are no vaccines or specific antiviral treatments available for HeV infection of humans [8,9]. However, a trial with 40 healthy human volunteers showed that the monoclonal antibody m102.4, which neutralises HeV and the closely related Nipah virus (NiV), was safe and well tolerated indicating great potential for future prevention and treatment of henipavirus infections [10].

Distribution of HeV and NiV are currently separated by the biogeographic region known as Wallacea, with presence of HeV confirmed in bats in Australia and Papua New Guinea, while presence of NiV has been confirmed in Bangladesh, Malaysia, Cambodia, Sumatra, Java and Borneo [11]. Due to expansion and movement of bat home-ranges, a diagnostic test with high sensitivity and specificity that enables early detection of HeV infection is needed for both surveillance and disease control. To date diagnosis of HeV has primarily relied on reverse transcription polymerase chain reaction (RT-PCR) testing [12]. As a BSL-4 pathogen, testing requires transport of samples from regional settings to centres with diagnostic capacity [13]. Failure of timely HeV diagnosis can result in detrimental treatment delays impacting the Australian equine industry and health of farmers, stablehands, farriers, and veterinarians. An ideal HeV point-of-care test should be well suited for widespread screening according to the REASSURED criteria for diagnostic testing (Real-time connectivity, Ease of specimen collection and environmental friendliness, Affordable, Sensitive, Specific, User-friendly, Rapid and robust, Equipment free and Deliverable to end-users) [14,15]. One isothermal technology with potential to meet the REASSURED criteria is recombinase polymerase amplification (RPA), which does not require thermal cycling instrumentation for nucleic acid amplification and has been extensively used to detect zoonotic [[16], [17], [18], [19], [20], [21], [22], [23], [24]] and equine [25] pathogens. When combined with lateral flow detection (LFD) [23,24,[26], [27], [28], [29]] RPA offers a simple to use assay format that uses minimal equipment and is ideal for diagnostic point-of-care testing in resource-poor settings [30]. However, RPA and other emerging isothermal amplification technologies require prior nucleic acid purification, which is a lengthy multi-step process not suited to low-resource implementation, preventing full implementation in true point-of-care settings.

Here we describe a rapid HeV test that combines RPA-LFD with a unique sample preparation reagent, TNA-Cifer Reagent E, shown to inactivate and process Dengue virus serotypes 1–4 in clinically relevant matrices (blood, plasma and serum). We demonstrate TNA-Cifer Reagent E is also able to inactivate HeV, and can be combined with RPA-LFD to enable detection of HeV in as little as 30 min.

## 2 Methods

### 2.1 Plasmids and RNA template preparation

Plasmids containing either a HeV N gene fragment (MN062017.1, 694–993 nt) or NiV Nucleocapsid protein (N) gene fragment (JN808863.1, 694–993 nt) were obtained from Bioneer Pacific (Victoria, Australia).

To produce RNA templates, DNA plasmids containing either the HeV N gene or NiV N gene were linearized by restriction with Xho1 (New England Biolabs (Australia) Pty Ltd., Victoria, Australia), electrophoresed and purified (NucleoSpin® Gel and PCR Clean-up, Macherey-Nagel, Düren, Germany). RNA transcripts subsequently generated by in vitro transcription according to manufacturer's instructions (MEGAscript® T7 transcription kit, Invitrogen by Thermo Fisher Scientific Australia Pty Ltd., Victoria, Australia).

#### 2.1.1 Oligonucleotides

HeV-specific primers and nfo-probes targeting conserved regions of the N gene were designed manually according to criteria described by the manufacturer (TwistDX) using an alignment of 89 published sequences present in the Genbank database and are available on request. Primers and probes were tested in various combinations using synthetic RNA transcripts for optimization. Primers and probes for the RT-qPCR test were previously described [31].

### 2.2 Viruses, cell culture, virus culture, virus titre determination and mock samples

#### 2.2.1 Viruses

All viral strains used in this study are listed in Table 1. For inactivation, Nipah virus and Hendra virus stocks were sent to a commercial gamma-irradiator (Steritech, Dandenong Australia) where they were treated with 50 kGy gamma-irradiation for inactivation.

**Table 1:** Viral strains used in this study.

Virus	Abbreviation	Strain and source (if known)	GenBank accession number
Hendra virus	HeV	Australia/Horse/2008/Redlands	HM044317
Nipah virus	NiV_B_	Bangladesh/Human/2004/Rajbari, R1	AY988601
Nipah virus	NiV_M_	Malaysia/Human/99	AF212302
Dengue virus serotype 1	DENV-1	ET00.243	JN415499
Dengue virus serotype 2	DENV-2	ET00.300	JN568254
Dengue virus serotype 3	DENV-3	East Timor 2000	JN575566
Dengue virus serotype 4	DENV-4	ET00.288	JN575585
Japanese encephalitis virus	JEV	Nakayama	EF571853
Murray Valley encephalitis virus	MVEV	1–51	AF161266
yellow fever virus	YFV	17D	MT505351
West Nile virus	WNV_KUNJ_	Kunjin strain NSW 2011	JN887352
Zika virus	ZIKV	MR766	KX830960
Chikungunya virus	CHIKV	Mauritius 2006	MH229986

#### 2.2.2 Cell culture

Vero cells (Vero C1008) were obtained from ATCC. Vero cells were grown in Minimal Essential Medium (Gibco by Thermo Fisher Scientific Australia Pty Ltd., Victoria, Australia) containing 1× Antibiotic/Antimycotic solution (100 U/mL penicillin, 100 μg/mL streptomycin, 0.25 μg/mL Amphotericin B; Gibco by Thermo Fisher Scientific Australia Pty Ltd., Victoria, Australia), and 10% fetal calf serum (Gibco by Thermo Fisher Scientific Australia Pty Ltd., Victoria, Australia), designated MEM-10), at 37 °C and 5% CO2. *Aedes albopictus* clone C6/36 (ATCC CRL-1660) were obtained from the American Type Culture Collection. C6/36 cells were cultured in RPMI 1640 (Thermo Fisher Scientific Australia Pty Ltd., Victoria, Australia) with 5% heat-inactivated fetal bovine serum (Sigma-Aldrich, New South Wales, Australia), 2 mmol/L l-glutamine (Gibco by Thermo Fisher Scientific Australia Pty Ltd., Victoria, Australia) and 100 U/mL Penicillin, 100 μg/mL Streptomycin and 0.25 μg/mL Amphotericin B (Sigma-Aldrich, New South Wales, Australia), at 28 °C and 5% CO2. Before reaching confluency, Vero and C6/36 cells were trypsinized with 0.25% trypsin solution (Gibco by Thermo Fisher Scientific Australia Pty Ltd., Victoria, Australia) and resuspended in their corresponding fresh growth media before plating onto a new growth surface.

#### 2.2.3 Virus culture

For viral culture, HeV and NiV were propagated in Vero cells with a low MOI (multiplicity of infection; approximate MOI of 0.01) in T175 cm flasks. Virus containing supernatant was harvested at approximately 72 h, when significant CPE was visible (greater than 70% of cells in syncytia), stock clarified by centrifugation at 5000 ×*g* for 10 min and then stored at −80 °C until needed. Flavivirus strains and CHIKV were propagated to a concentration of 10^5^ to 10^7^ tissue culture infectious dose (TCID)_50_/mL in T25 culture flasks seeded with C6/36 cells in RPMI 1640 growth media as described above, with the exception that 2% FBS was used. 7 days post infection, 2 mL of TRI Reagent (Sigma-Aldrich, New South Wales, Australia) was added to the flask preparation and swirled over the cell area for 1–2 min. To prepare the inactivated viruses for extraction the culture supernatant was separated into a tube and centrifuged at 3000 *g* for 10 min at 4 °C to separate supernatant from cell pellet. The total RNA was extracted from the infected culture cell supernatant stocks using the TRI Reagent extraction protocol, resuspended in nuclease-free water and stored at −80 °C.

#### 2.2.4 Virus titre determination

Virus stocks of HeV and NiV were measured by standard TCID_50_ assay using Vero cells in a 96-well plate. A 10-fold dilution series was prepared and 4 samples per dilution were plated. Wells were scored as CPE positive on day 4 post-inoculation. Titres were calculated using the Reed and Muench method [32].

#### 2.2.5 HeV inactivation

Viable HeV recovery was determined by incubating HeV containing samples with sample preparation reagent (TNA-Cifer Reagent E; BioCifer, Buderim, Australia). The mixtures were then added to wells containing Vero E6 cells (approximately 70% confluent) and incubated for 7 days. Individual wells were scored as either positive or negative for the presence of cytopathic effect (CPE) typical of HeV (syncytia). Wells showing no evidence of HeV CPE had 200 μL supernatant removed and added to a new 6-well plate with fresh Vero cells (blind passage), again incubated for 7 days before scoring for HeV CPE. This blind passaging of negative samples was done for two additional passages.

### 2.3 RNA purification and isolation

RNA from viral stocks was purified using TRIzol™ (Invitrogen by Thermo Fisher Scientific Australia Pty Ltd., Victoria, Australia) or with a silica-based kit (NucleoSpin RNA Virus Mini kit, Macherey-Nagel, Düren, Germany), following the manufacturer's instructions (20 μL sample was eluted in 20 μL nuclease-free water). For Taqman PCR, RNA was extracted from HeV stocks using a MagMAX™ Viral RNA Isolation Kit (Thermo Fisher Scientific Australia Pty Ltd., Victoria, Australia) following manufacturer's instructions. RNA was eluted in a final volume of 60 μL. All purified RNA was stored in aliquots at −80 °C.

### 2.4 Taqman PCR

TaqMan qPCR was performed using the AgPath-ID one-step reverse transcription-PCR kit (Thermo Fisher Scientific Australia Pty Ltd., Victoria, Australia), targeting the N gene of HeV or NiV as previously described [31]. Copy numbers were calculated using a previously derived formula from a standard curve generated using a synthetic DNA fragment with known copy number across a 10 fold dilution series to less than 1 copy per reaction.

### 2.5 Rapid Hendra test

Rapid sample processing was achieved by mixing samples containing N gene RNA or cultured HeV with sample preparation reagent (TNA-Cifer Reagent E; BioCifer, Brisbane, Australia) at a ratio of 1:1 and incubated for 2 min on ice. The samples were then diluted 1:5 in nuclease-free water.

HeV RT-RPA-LFD assay amplified the N gene using the TwistAmp™ exo kit (TwistDX, Cambridge, United Kingdom) and HybriDetect lateral flow strips (Milenia Biotec, Giessen, Germany). In brief, RPA reaction mixes consisted of 1× rehydration buffer, 1/5 rehydrated lyophilized pellet, forward primer (420 nM), reverse primer (420 nM), probe (120 nM), Ribolock (10 U), Endonuclease IV (2 U; New England Biolabs, Victoria, Australia), and Moloney Murine Leukemia virus reverse transcriptase (mMLV, 40 U), magnesium acetate (14 mM) and template (1 μL) in a final 10 μL reaction volume. Following incubation at 39 °C for 20 min the reaction mixes were transferred to pre-activated lateral flow strips [33], which were then placed in running buffer [24] for 5 min. The lateral flow strips were firstly analysed by eye. The appearance of two lines (i.e., a test line along and the control line) was scored a positive result for presence of HeV. The absence the test line but presence of control line was scored as a negative result. To provide quantitative data lateral flow strips were also scanned with an Epson Perfection V39 Flatbed Scanner (Epson, New South Wales, Australia) and resulting images analysed using ImageJ software (National Institutes of Health, MD, USA) as previously described [24,28].

### 2.6 Sensitivity and specificity testing

Analytical sensitivity for the HeV RT-RPA-LFD assays was assessed using 10-fold serial dilutions of synthetic RNA transcripts containing HeV N gene fragments. Analytical sensitivity testing of the rapid Hendra tests was determined using titred virus diluted 10-fold in a viral transport medium (VTM; Minimal Essential Medium containing 0.1% bovine serum albumin, 500 U/mL Penicillin, 500 μg/mL Streptomycin and 2500 μg/mL Fungizone (Thermo Fisher Scientific Australia Pty Ltd., Victoria, Australia). Analytical specificities for HeV RT-RPA-LFD assay were determined using kit-purified RNA of NiV_B_ (1.00 × 10^6^ TCID_50_/mL), NiV_M_ (1.00 × 10^6^ TCID_50_/mL) DENV-1 (ET00.243, 1.35 × 10^6^ TCID_50_/mL), DENV-2 (New Guinea C, 1.81 × 10^5^ TCID_50_/mL), DENV-3 (ET00.209, 2.89 × 10^5^ TCID_50_/mL) and DENV-4 (ET00.288, 3.31 × 10^6^ TCID_50_/mL) isolates. In addition, specificity testing used TRIzol-purified RNA of cell culture supernatants from CHIKV, JEV, MVEV, YFV, WNV_KUNJ_, and ZIKV, at high RNA concentrations (undiluted). Analytical specificities for HeV RT-RPA-LFD assay were also evaluated against a range of previously screened kit-purified DNA extracts from equine cultured viral (Equine herpesvirus (EHV)-1, −2, −4 and − 5) and bacterial (*Streptococcus equi* subspecies *zooepidemicus*, *S. equi equi*, *S. dysgalactiae* subspecies *equisimilis* and *Chlamydia psittaci*) pathogens.

## 3 Results

### 3.1 Analytical sensitivity of the HeV RT-RPA-LFD assay using synthetic RNA

**Fig. 1:**
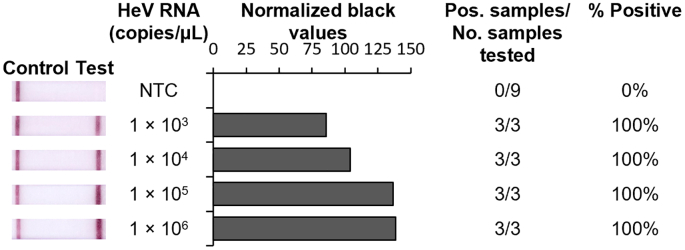
Analytical sensitivity of HeV RT-RPA-LFD assay testing synthetic HeV RNA. Sensitivity testing used 10-fold serially diluted synthetic RNA from HeV. Images of lateral flow strips with two lines (control and test line) indicated the sample is positive detecting HeV-specific RNA, and single control lines indicated a valid reaction with negative sample, such as nuclease-free water as no template control (NTC) (left). Normalised pixel densities (normalised black values) from the lateral flow strips displayed (middle). Positive (Pos.) samples compared to number (No.) of samples tested at that dilution was used to calculate the percentage of positive tests performed at that dilution (right).

To develop rapid, low-resource tests for detection of HeV, we designed RT-RPA assays that targeted a conserved region within the HeV Nucleocapsid protein (N) gene previously used for development of RT-PCR assays [31,34,35]. The sensitivity of the assay was first assessed using synthetic RNA. Under these conditions our HeV RT-RPA-LFD assay was reproducibly able to detect 1000 copies/μL of synthetic HeV RNA (Fig. 1).

### 3.2 Analytical specificity of the HeV RT-RPA-LFD assay using purified virus RNA

**Fig. 2:**
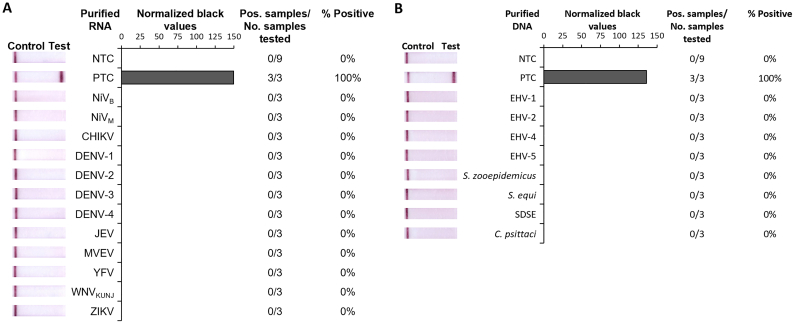
Analytical specificity of HeV RT-RPA-LFD assay testing purified RNA of other viruses. Specificity testing used kit-purified RNA of Nipah virus Bangladesh (NiV_B_) and Malaysia (NiV_M_) strains, dengue virus (DENV-1, −2, −3, and − 4), and TRIzol-purified RNA of chikungunya virus (CHIKV), Japanese encephalitis virus (JEV), Murray Valley encephalitis virus (MVEV), yellow fever virus (YFV), West Nile virus (subtype Kunjin; WNV_KUNJ_), and Zika virus (ZIKV), kit-purified DNA of Equine herpesviruses (EHV-1, EHV-2, EHV-4, and EHV-5), *Streptococcus equi* subspecies *zooepidemicus* (*S. zooepidemicus*), *Streptococcus equi* subspecies *equi* (*S. equi*), *Streptococcus dysgalactiae* subspecies *equisimilis* (*SDSE*) and *Chlamydia psittaci* (*C. psittaci*), as well as, synthetic HeV RNA (10^6^ copies/μL) as positive template control (PTC). Images of lateral flow strips with two lines (control and test line) indicated the sample is positive detecting HeV-specific RNA, and single control lines indicated a valid reaction with negative sample, such as nuclease-free water as no template control (NTC) (left). Normalised pixel densities (normalised black values) from the lateral flow strips displayed (middle). Positive (Pos.) samples compared to number (No.) of samples tested of that purified viral RNA sample was used to calculate the percentage of positive tests performed with that purified viral RNA sample (right). (For interpretation of the references to colour in this figure legend, the reader is referred to the web version of this article.)

The analytical specificity of the optimized HeV RT-RPA-LFD assay was assessed using purified RNA from Nipah virus, chikungunya virus and a range of other flaviviruses (Fig. 2A), and DNA from Equine herpesviruses (EHV-1, EHV-2, EHV-4, and EHV-5), *Streptococcus equi* subspecies *zooepidemicus*, *S. equi equi*, *S. dysgalactiae* subspecies *equisimilis* and *Chlamydia psittaci* (Fig. 2B). Apart from HeV no other pathogens were detected by the assay.

### 3.3 Sample preparation reagent inactivates HeV

A rapid low-resource test requires a simple sample preparation procedure, and for this we wanted to trial the rapid sample preparation reagent, TNA-Cifer Reagent E. However, since HeV is a BSL-4 agent, we first wanted to trial the conditions at which TNA-Cifer Reagent E could inactivate Hendra virus, as has been observed for DENV-1 and NiV ( [36,37]; and also SARS-CoV-2, unpublished). Our results showed that at a 1:1 sample:reagent ratio, HeV was inactivated within 10 min at room temperature, and this was confirmed by serial passaging of the virus. However, at lower ratio of reagent to sample HeV was not inactivated (Table 2). Subsequent testing demonstrated that a shorter 2 min incubation at a 1:1 sample:reagent ratio inactivated the virus completely (Table 2).

**Table 2:** Inactivation of HeV by sample preparation reagent.

Sample to sample preparation reagent ratio	Incubation time (min)	CPE positive wells		
		P1	P2	P3
5:1	10	3/3	–	–
2:1	10	1/3	1/3	1/3
1:1	10	0/3	0/3	0/3
1:1 control (sample to PBS)	10	3/3	–	–
1:1	2	0/3	0/3	0/3
1:1 control (sample to PBS)	2	3/3	–	–

Individual wells were scored as either positive or negative for the presence of cytopathic effect (CPE) typical of HeV (syncytia) and passaged blind twice (P1, P2, P3) if at least one of three wells showed no evidence of HeV CPE. Number of CPE positive wells compared to total number of wells for each condition.

### 3.4 Detection of HeV in viral transport medium

**Fig. 3:**
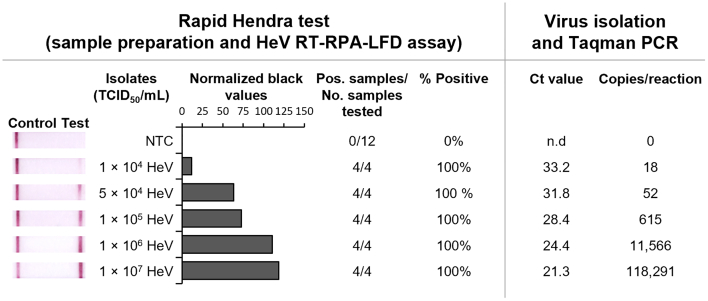
Detection of HeV isolate in viral transport medium with the rapid Hendra test. Viral transport medium containing gamma-irradiated HeV isolate was diluted in viral transport medium and analysed with the rapid Hendra test (left) or viral RNA was isolated using a kit and then assessed by Taqman PCR (right). Rapid Hendra test: Images of lateral flow strips with control lines (all samples including viral transport medium as no template control (NTC)) and test lines (positive samples); Normalised pixel densities (normalised black values) from the lateral flow strips displayed; Positive (Pos.) samples compared to number (No.) of samples tested at that dilution was used to calculate the percentage of positive tests performed at that dilution. Virus isolation and Taqman PCR: Comparative PCR testing cycle threshold (Ct) and calculated copy number (Copies/reaction).

Using the ratio of TNA-Cifer Reagent E shown to inactivate cultured HeV, we trialled creation of a rapid HeV test by combining rapid sample preparation with RPA-LFD. Optimisation testing indicated that sample preparation by mixing sample:reagent 1:1, followed by a 1/6 dilution in water, enabled samples to be subsequently detected by RPA-LFD (data not shown; higher or lower dilution resulted in a reduction of band intensity). We trialled the optimized rapid HeV test protocol using gamma-irradiated HeV spiked into a viral transport medium. Comparative testing utilized a standard RNA purification procedure to isolate viral RNA and Taqman PCR for HeV detection [31]. Our rapid Hendra test detected a minimum of 10,000 TCID_50_/mL of HeV, which was calculated by Taqman PCR to be equivalent to 18 copies/reaction (Fig. 3).

## 4 Discussion

Spillover events of HeV continue to pose a substantial public health threat in Australian urban and peri-urban areas [3,4,38]. Current ‘gold-standard’ RT-PCR for the detection of HeV remains prominent within the equine and veterinary industry. However, this technique comes with several drawbacks that limits its use, particularly in resource-poor settings. In this study, we developed a rapid HeV test in a simple low-resource format, facilitating HeV virus detection within 30 min. We demonstrated the assay was able to detect as little as 1000 copies/μL synthetic HeV RNA, and did not cross react with any viruses. Moreover the assay was able to detect gamma-irradiated HeV in samples mimicking in-field collection conditions with a sensitivity of 10,000 TCID_50_/mL, equivalent of 18 copies per reaction.

The majority of previous studies have used RT-PCR for the detection of HeV RNA. While no in-field rapid test has been evaluated, one promising RT-LAMP-LFD test has been reported, testing nasal swabs of experimentally infected horses (*n* = 3). Results indicated a detection limit comparable to Taqman RT-PCR cycle threshold (Ct) values [39], however, the PCR test required kit-purified viral RNA and requires samples to be shipped to a centralised containment facility for sample processing and thermocycling, with the testing time taking approximately four hours. Our test showed 100% congruence with Taqman PCR results and has a simple and rapid sample processing procedure eliminates the need for laborious RNA purification procedures.

As HeV is a BSL-4 pathogen, activities including virus isolation prior to RT-PCR testing are currently required to be performed in a BSL-4 laboratory. One key advantage of our sample processing method is the inactivation of virus in the first step of the detection procedure. Similar results have been obtained for NiV_B_ and NiV_M_ strains, revealing complete inactivation occurred as early as 2 min [36]. The sample processing method thus provides a pathway for safer point-of-care testing, or safer testing in low-resource environments that do not have full biohazard facilities. By combining with RT-RPA and LFD we enable easy to read results with potential for rapid diagnosis in the field. Our test could help improve biosecurity responses, enabling better management of animal and human health, providing safe and rapid sample processing combined with sensitive and accurate detection of early HeV infection.

Our primers and probe were designed to detect all Hendra virus isolates by targeting a conserved region of the HeV N gene. During the course of our study a second genotype of Hendra virus was published [40]. RPA can tolerate some mutations between primer and probe sequences [41], and bioinformatic analysis indicates HeV genotype 2 has only 4 mutations in each of our primer and probes binding regions, suggesting that our test may detect HeV genotype 2 strains, although this needs to be confirmed experimentally. Further testing should also be performed to trial our test during in-field testing to show operational suitability for veterinarians to screen for HeV, including EDTA blood, serum, and swabs of mucosal surfaces (nasal, ocular, oral, rectal). The effect of nucleases in the serum and viral transport medium (VTM) should also be considered, although we note that TNA-Cifer Reagent E can effectively neutralise up to 150 μg/mL RNase A (unpublished data). In addition, further HeV inactivation testing should consider the minimal TNA-Cifer Reagent E concentration, as well as the effect of blood, serum, sub-optimal sample matrices, or other potential inhibitors of inactivation before field trialling tests. High precautions must be taken from sampling through to result readout and clean-up to minimize risk of exposure to any zoonotic diseases. Adequate personal protective equipment (PPE) including correct donning and doffing procedures, correct storage, maintenance, cleaning, disinfection, and disposal of PPE will remain paramount to operator safety.

## 5 Conclusions

Our rapid Hendra test combines a simple sample processing procedure that inactivates the pathogen, isothermal amplification of RNA using RT-RPA, and a simple field-friendly detection using lateral flow strips. The test was able to detect as few as 10,000 TCID_50_/mL, equivalent of 18 RNA copies per reaction. Our test has several advantages compared to Taqman PCR, such as a simple workflow, rapid sample processing and turnaround time (30 min from sample preparation to detection), minimal equipment needs, and improved safety for testing outside of a laboratory through the inactivation of virus during the sample preparation step, with the potential of testing in low-resource settings to provide results directly to veterinarians or government workers performing the test. These specifications indicate our test is able to meet the REASSURED criteria for diagnostic testing [14,15]. The low-resource format of our rapid Hendra test could improve biosecurity response and enhance diagnostic testing capacity for the Australian equine industry.

## Funding

This study was supported by the DMTC Limited (VIC, Australia) Medical Countermeasures Program [Project 10.75]; BioCifer Pty. Ltd. (QLD, Australia); the Queensland Government, Department of Science, Information Technology and Innovation, Australia; and public donations to the 10.13039/501100001796University of the Sunshine Coast (QLD, Australia). This work was also supported, in part, by the 10.13039/100000865Bill & Melinda Gates Foundation [OPP1140133]. Under the grant conditions of the Foundation, a Creative Commons Attribution 4.0 Generic License has already been assigned to the Author Accepted Manuscript version that might arise from this submission.

## CRediT authorship contribution statement

**N.M. Pollak:** Conceptualization, Data curation, Formal analysis, Investigation, Methodology, Project administration, Resources, Supervision, Validation, Visualization, Writing – original draft, Writing – review & editing. **G.A. Marsh:** Data curation, Formal analysis, Investigation, Methodology, Resources, Validation, Visualization, Writing – review & editing. **M. Olsson:** Data curation, Formal analysis, Investigation, Methodology, Validation, Visualization, Writing – review & editing. **D. McMillan:** Conceptualization, Formal analysis, Funding acquisition, Methodology, Project administration, Resources, Supervision, Writing – review & editing. **J. Macdonald:** Conceptualization, Formal analysis, Funding acquisition, Methodology, Project administration, Resources, Supervision, Writing – review & editing.

## Declaration of Competing Interest

Pollak NM is a funded post-doctoral research scientist for DMTC Ltd., Australia. Macdonald J is a Project Leader for DMTC Ltd., Australia. Macdonald J is a co-founder, shareholder, and director of BioCifer Pty. Ltd., who has licensed the technology. All other authors declare no competing interest.

## Data Availability

The original data generated for this study has been included in the article; further inquiries can be directed to the corresponding author/s.

## References

[1] Government Q. (2021). Summary of Hendra Virus Incidents in Horses: Queensland Government. https://www.business.qld.gov.au/industries/service-industries-professionals/service-industries/veterinary-surgeons/guidelines-hendra/incident-summary.

[2] Government Q. (2018). Hendra Virus. https://www.business.qld.gov.au/industries/farms-fishing-forestry/agriculture/livestock/animal-welfare/pests-diseases-disorders/hendra-virus.

[3] Plowright R.K., Foley P., Field H.E., Dobson A.P., Foley J.E., Eby P. (2011). Urban habituation, ecological connectivity and epidemic dampening: the emergence of Hendra virus from flying foxes (Pteropus spp.). Proc. Biol. Sci..

[4] McFarlane R., Becker N., Field H. (2011). Investigation of the climatic and environmental context of Hendra virus spillover events 1994-2010. PLoS One.

[5] Government N.S.W. (2022). Summary of Human Cases of Hendra Virus Infection. https://www.health.nsw.gov.au/Infectious/controlguideline/Pages/hendra-case-summary.aspx.

[6] Prevention CfDCa (2021). Hendra Virus Disease: Signs and Symptoms. https://www.cdc.gov/vhf/hendra/index.html.

[7] Marsh G.A., Haining J., Hancock T.J., Robinson R., Foord A.J., Barr J.A. (2011). Experimental infection of horses with Hendra virus/Australia/horse/2008/Redlands. Emerg. Infect. Dis..

[8] Halpin K., Graham K., Durr P.A. (2021). Sero-monitoring of horses demonstrates the Equivac((R)) HeV Hendra virus vaccine to be highly effective in inducing neutralising antibody titres. Vaccines (Basel).

[9] Organization WH (2008). Hendra virus infection - Treatment. https://www.who.int/health-topics/hendra-virus-disease#tab=tab_3.

[10] Playford E.G., Munro T., Mahler S.M., Elliott S., Gerometta M., Hoger K.L. (2020). Safety, tolerability, pharmacokinetics, and immunogenicity of a human monoclonal antibody targeting the G glycoprotein of henipaviruses in healthy adults: a first-in-human, randomised, controlled, phase 1 study. Lancet Infect. Dis..

[11] Breed A.C., Meers J., Sendow I., Bossart K.N., Barr J.A., Smith I. (2013). The distribution of henipaviruses in Southeast Asia and Australasia: is Wallace’s line a barrier to Nipah virus?. PLoS One.

[12] (OIE) WOfAH (2018). Manual of Diagnostic Tests and Vaccines for Terrestrial Animals.

[13] Yuen K.Y., Fraser N.S., Henning J., Halpin K., Gibson J.S., Betzien L. (2021). Hendra virus: epidemiology dynamics in relation to climate change, diagnostic tests and control measures. One Health.

[14] Kosack C.S., Page A.L., Klatser P.R. (2017). A guide to aid the selection of diagnostic tests. Bull. World Health Organ..

[15] Land K.J., Boeras D.I., Chen X.S., Ramsay A.R., Peeling R.W. (2019). REASSURED diagnostics to inform disease control strategies, strengthen health systems and improve patient outcomes. Nat. Microbiol..

[16] Euler M., Wang Y., Otto P., Tomaso H., Escudero R., Anda P. (2012). Recombinase polymerase amplification assay for rapid detection of Francisella tularensis. J. Clin. Microbiol..

[17] Kissenkotter J., Hansen S., Bohlken-Fascher S., Ademowo O.G., Oyinloye O.E., Bakarey A.S. (2018). Development of a pan-rickettsial molecular diagnostic test based on recombinase polymerase amplification assay. Anal. Biochem..

[18] Coertse J., Weyer J., Nel L.H., Markotter W. (2019). Reverse transcription recombinase polymerase amplification assay for rapid detection of canine associated rabies virus in Africa. PLoS One.

[19] Pang Y., Cong F., Zhang X., Li H., Chang Y.F., Xie Q. (2021). A recombinase polymerase amplification-based assay for rapid detection of chlamydia psittaci. Poult. Sci..

[20] Yehia N., Eldemery F., Arafa A.S., Abd El Wahed A., El Sanousi A., Weidmann M. (2021). Reverse transcription recombinase polymerase amplification assay for rapid detection of avian influenza virus H9N2 HA gene. Vet. Sci..

[21] Faye M., Seye T., Patel P., Diagne C.T., Diagne M.M., Dia M. (2022). Development of real-time molecular assays for the detection of Wesselsbron virus in Africa. Microorganisms..

[22] Jiang X., Zhu L., Zhan D. (2022). Development of a recombinase polymerase amplification assay for rapid detection of Streptococcus suis type 2 in nasopharyngeal swab samples. Diagn. Microbiol. Infect. Dis..

[23] Jimenez-Coello M., Shelite T., Castellanos-Gonzalez A., Saldarriaga O., Rivero R., Ortega-Pacheco A. (2018). Efficacy of recombinase polymerase amplification to diagnose Trypanosoma cruzi infection in dogs with cardiac alterations from an endemic area of Mexico. Vector Borne Zoonotic Dis..

[24] Li J., Pollak N.M., Macdonald J. (2019). Multiplex detection of nucleic acids using recombinase polymerase amplification and a molecular colorimetric 7-segment display. ACS Omega.

[25] Lei R., Wang X., Zhang D., Liu Y., Chen Q., Jiang N. (2020). Rapid isothermal duplex real-time recombinase polymerase amplification (RPA) assay for the diagnosis of equine piroplasmosis. Sci. Rep..

[26] Bonnet E., van Jaarsveldt D., Burt F.J. (2022). Rapid reverse transcriptase recombinase polymerase amplification assay for flaviviruses using non-infectious in vitro transcribed RNA as positive controls. J. Virol. Methods.

[27] Li T.T., Wang J.L., Zhang N.Z., Li W.H., Yan H.B., Li L. (2019). Rapid and visual detection of Trichinella Spp. using a lateral flow strip-based recombinase polymerase amplification (LF-RPA) assay. Front. Cell. Infect. Microbiol..

[28] James A.S., Todd S., Pollak N.M., Marsh G.A., Macdonald J. (2018). Ebolavirus diagnosis made simple, comparable and faster than molecular detection methods: preparing for the future. Virol. J..

[29] Ahmed M., Pollak N., Hugo L., van den Hurk A., Hobson-Peters J., Macdonald J. (2022). Rapid Molecular Assays for the Detection of the Four Dengue Viruses in Infected Mosquitoes [Version 2; Peer Review: 2 Approved]. Gates Open Res..

[30] Sadeghi P., Sohrabi H., Hejazi M., Jahanban-Esfahlan A., Baradaran B., Tohidast M. (2021). Lateral flow assays (LFA) as an alternative medical diagnosis method for detection of virus species: the intertwine of nanotechnology with sensing strategies. Trends Anal. Chem..

[31] Feldman K.S., Foord A., Heine H.G., Smith I.L., Boyd V., Marsh G.A. (2009). Design and evaluation of consensus PCR assays for henipaviruses. J. Virol. Methods.

[32] Reed L.J., Muench H. (1938). A simple method of estimating fifty percent endpoints. Am. J. Epidemiol..

[33] Rames E.K., Macdonald J. (2019). Rapid assessment of viral water quality using a novel recombinase polymerase amplification test for human adenovirus. Appl. Microbiol. Biotechnol..

[34] Wright P.J., Crameri G., Eaton B.T. (2005). RNA synthesis during infection by Hendra virus: an examination by quantitative real-time PCR of RNA accumulation, the effect of ribavirin and the attenuation of transcription. Arch. Virol..

[35] Guillaume V., Lefeuvre A., Faure C., Marianneau P., Buckland R., Lam S.K. (2004). Specific detection of Nipah virus using real-time RT-PCR (TaqMan). J. Virol. Methods.

[36] Pollak N.M., Ollson M., Marsh G.A., Macdonald J., McMillan M. (2023). Evaluation of three rapid low-resource molecular tests for Nipah virus. Front. Microbiol..

[37] Pollak N.M., Olsson M., Ahmed M., Tan J., Lim G., Setoh Y.X. (2023). Rapid Diagnostic Tests for the Detection of the Four Dengue Virus Serotypes in Clinically Relevant Matrices. Microbiol. Spectr..

[38] Plowright R.K., Eby P., Hudson P.J., Smith I.L., Westcott D., Bryden W.L. (2015). Ecological dynamics of emerging bat virus spillover. Proc. Biol. Sci..

[39] Foord A.J., Middleton D., Heine H.G. (2012). Hendra virus detection using loop-mediated isothermal amplification. J. Virol. Methods.

[40] Wang J., Anderson D.E., Halpin K., Hong X., Chen H., Walker S. (2021). A new Hendra virus genotype found in Australian flying foxes. Virol. J..

[41] Ahmed M., Pollak N.M., Devine G.J., Macdonald J. (2022). Detection of a single nucleotide polymorphism for insecticide resistance using recombinase polymerase amplification and lateral flow dipstick detection. Sensors Actuators B Chem..

